# Small Bowel Obstruction Secondary to Laparoscopic Adjustable Gastric Band Connecting Tube Intertwinement Within the Mesentery: A Case Report

**DOI:** 10.7759/cureus.39945

**Published:** 2023-06-04

**Authors:** Kapish Sharma, Sara Arfan, Sri Sai Prasanna Thota, Chukwuma Agbasi, Laiba Khan, Larab Naqvi, Frederick Tiesenga

**Affiliations:** 1 General Surgery, Windsor University School of Medicine, Cayon, KNA; 2 General Surgery, Washington University of Health and Science, San Pedro, BLZ; 3 General Surgery, Avalon University School of Medicine, Willemstad, CUW; 4 General Surgery, Community First Medical Center, Chicago, USA

**Keywords:** research in emergency medicine, gi radiology, obesity, bariatric surgery complications, small bowel obstruction, laparoscopic banding, adhesions

## Abstract

Laparoscopic adjustable gastric banding (LAGB) is a technique used for the surgical management of morbid obesity. This report illustrates the case of a 46-year-old African American woman who presented with a rare case of small bowel obstruction (SBO) two years post-LAGB placement. SBO, in this case, was a result of LAGB connecting tube intertwinement within the mesentery, accompanied by adhesions. The patient was diagnosed clinically and radiologically by computed tomography (CT) scan, which showed high-grade SBO. Initially, an exploratory laparoscopy was conducted, which soon transformed into an exploratory laparotomy when the cause of obstruction was seen to be the intertwinement of the connecting tube of the gastric band with the mesentery. With the rise of bariatric procedures to combat the epidemic of obesity in American society, this rare complication secondary to one of the most widely performed procedures beckons the attention of bariatric surgeons, emergency personnel, and device manufacturers.

## Introduction

In the field of bariatric procedures, the incidence of small bowel obstruction (SBO) presents as one of the most common surgical emergencies. Approximately, 75% of small bowel obstructions are due to intra-abdominal adhesions, usually as a consequence of previous surgical interventions [1], with a 20% rate of recurrence [2]. Laparoscopic adjustable gastric banding (LAGB), commonly known as a lap band, is a relatively simple and common bariatric procedure used for the long-term augmentation of weight management strategies in morbidly obese patients [3]. Morbid obesity is defined as BMI ≥ 40 kg/㎡, as well as BMI between 35 and 39.9 kg/㎡ when significant obesity-related health conditions are present [4]. The latest criteria for bariatric surgery illustrates that bariatric surgery is recommended for patients with BMI ≥ 35 kg/㎡, with or without the presence of accompanying co-morbid conditions, as well as in patients of Asian descent with a BMI ≥ 27.5 kg/㎡. It should be considered for patients with metabolic disease with a BMI of 30-34.9 kg/㎡. It serves to curtail oral calorie consumption via the mechanism of reducing mechanical stomach volume through the placement of an adjustable band around the stomach [5]. The gastric band is positioned around the top portion of the stomach during surgery and fixed in place with sutures or clips. The access port is then put in place beneath the skin and sutured. To link the gastric band to the access port, the connecting tube is tunneled under the skin. The LAGB device's adjustability enables gradual tightening or loosening of the band's restriction. The size of the opening created by the gastric band can be changed to manipulate how much food the stomach can contain, by injecting or withdrawing saline solution through the access port. It's worth noting that different manufacturers may use different designs and parts for their LAGB devices [6]. In order to achieve a scrupulous understanding of this case, it is important to be familiar with the anatomy of the LAGB device:

Gastric band: A silicone ring that is wrapped around the upper portion of the stomach is used to create a small pouch in the stomach. The intention is to limit how much food the stomach can hold, resulting in the sensation of fullness from smaller portions of food. Changes in the level of restriction are achievable, thanks to the band's adjustability.

Access port: During the LAGB procedure, an access port is placed in the abdominal wall beneath the skin, with a thin tube linking it to the gastric band. Saline solution can be injected into or removed from the access port to change how tight the band is. A needle placed into the access port can be used to perform this in the doctor's office.

Connecting tube: Saline solution can be transferred while making modifications thanks to the connecting tube that goes between the access port and the gastric band.

Injection port: The connection tube joins the injection port, a small, self-sealing component, to the access port. It enables the surgeon or medical professional to insert a needle into the access port to modify the band [6-8].

We present the rare case of a 46-year-old African American woman presenting with SBO due to connecting tube intertwinement within the intestinal mesentery accompanied by intestinal adhesions two years after receiving the LAGB procedure.

## Case presentation

**Figure 1:**
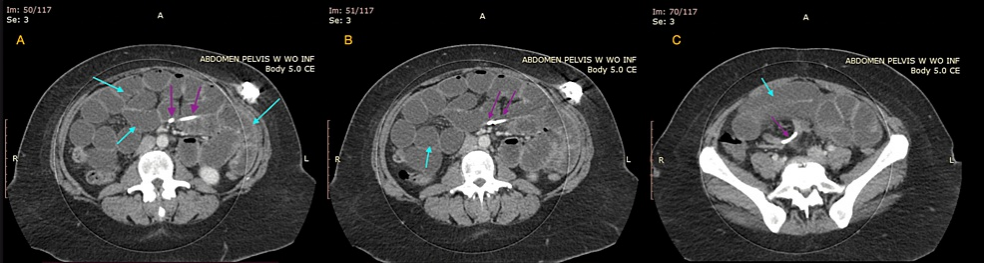
Axial non-contrast abdominal CT demonstrating dilated small bowel loops Non-contrast CT of the abdomen in the axial plane (A-C) demonstrating multiple, dilated small bowel loops (blue arrows), along with the path taken by the LAGB connecting tube to intertwine with mesenteric circulation (purple arrows) LAGB: laparoscopic adjustable gastric banding

**Figure 2:**
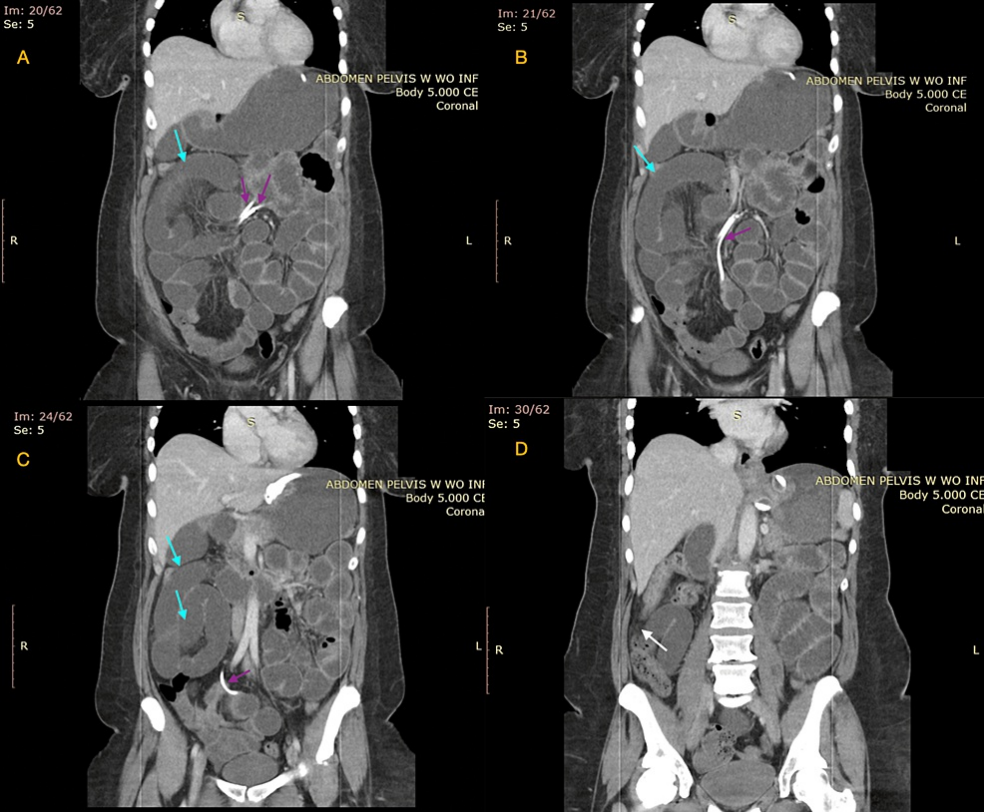
Abdominal non-contrast CT in the coronal plane (A-D) demonstrating multiple, dilated small bowel loops Figures A-C demonstrate the path taken by the LAGB connecting tube to intertwine with the mesenteric circulation (purple arrows), as well as multiple loops of dilated small bowel (blue arrows). Figure D demonstrates the transition point between the small bowel obstruction and the normal distal bowel (white arrow). LAGB: laparoscopic adjustable gastric banding

We present the case of a 46-year-old African American woman presenting to the emergency department complaining of abdominal pain, nausea, and vomiting. She was referred from another facility after her CT scan revealed a high-grade SBO (Figures 1, 2).

The patient’s past medical history was significant for morbid obesity with BMI > 40 kg/㎡ and no accompanying metabolic disorders, for which she underwent a LAGB placement procedure two years ago, using the LapBand device (INAMED Health, Santa Barbara, CA). On admission, she reported excruciating pain that “hurts like the worst pain imaginable." Her abdominal pain and distension were accompanied by nausea and aversion to food. The distension became apparent after she stopped having bowel movements.

The patient was alert, orientated, and in apparent distress. The patient did not use tobacco, alcohol, or any illicit drugs. Family history was non-contributory. Her vitals on admission revealed a temperature of 98.5 F, pulse of 92 bpm, respiratory rate of 20 bpm, blood pressure of 161/94 mmHg, and oxygen saturation of 98% on room air. BMI was 39.06 kg/m². A complete blood count (CBC) differential was significant for an elevated white blood count (WBC) of 14.3 K/mm^3^ (normal: 3.7-10.5 K/mm3). The basic metabolic panel, liver function tests, and lactic acid were unremarkable. On physical examination, the abdomen was soft, diffusely tender, and distended. Rebound tenderness and hyperactive bowel sounds were appreciated.

Evidence from history, physical exam, and imaging dictated that the patient would undergo an exploratory laparoscopy with adhesiolysis, which, upon visualization, transformed into an exploratory laparotomy with adhesiolysis, enteroclysis and release of SBO along with the revision of the laparoscopic gastric band tubing. Intraoperatively, the patient had tense, discolored, dilated small bowel loops that had entirely collapsed distally. Upon visualization, it was established that the length of the connecting tube was the root cause of its intertwinement with the mesentery, leading to the development of SBO. The connecting tube was transected to relieve the obstruction and multiple thick adhesions between the loops of the small bowel were meticulously lysed. A segment of tubing from the junction of the band was unable to be removed, however, the connection of the port site to the previous transected piece of the tube was re-established, which allowed the continuity of the lap band system. Due to no abnormality or complications attributed to the LapBand device itself or its placement, the device was not removed. Hemostasis was ensured and the Jackson-Pratt (JP) surgical drain was placed owing to the nature of the procedure being an open exploratory laparotomy. There were no intraoperative complications.

**Figure 3:**
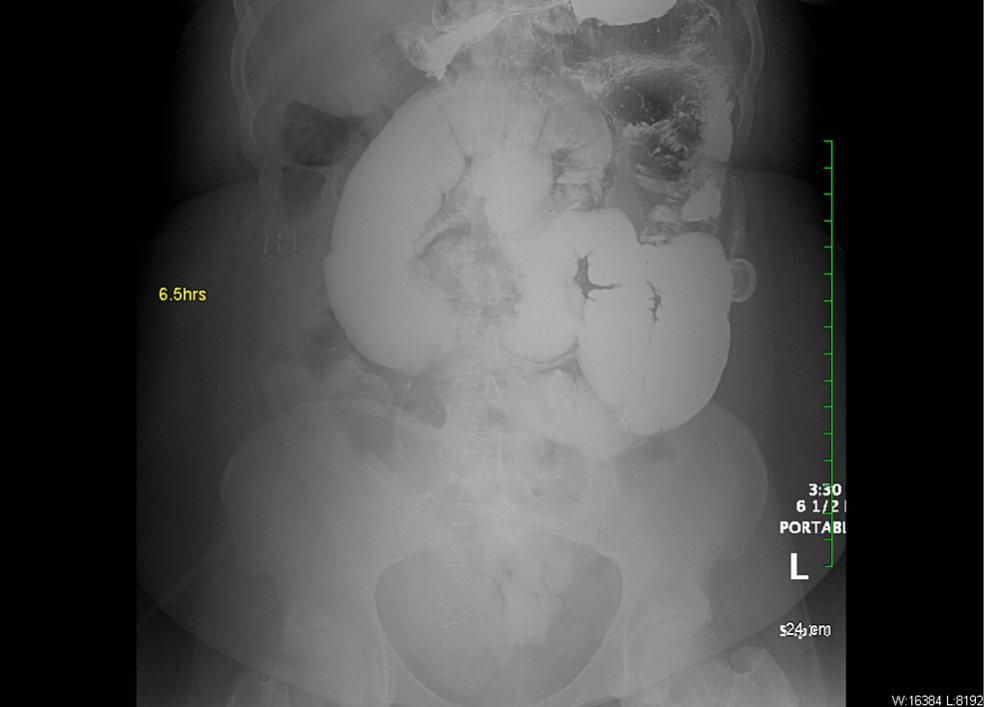
Small bowel follow-through at 6.5 hours demonstrating gas-filled small and large bowel loops, without any obstruction

The patient’s postoperative course was complicated by postop ileus and jejunal enteritis. The patient’s first bowel movement was on postoperative Day 7. Leukocytosis was noted on postoperative Day 1 and shortly resolved before antibiotic administration. Significant abdominal pain exacerbated with ambulation was reported on postoperative Day 5, at which point a kidney, ureter, and bladder (KUB) X-ray and small bowel follow-through study were ordered. Ingestion of contrast for the latter study resulted in emesis. Results from the small bowel follow-through study revealed a delayed transit (6.5 to 10.5 hours; normal: 30 minutes to 3 hours) and gas-filled small and large bowel loops without any obstruction to the antegrade flow of contrast into the proximal small bowel (Figure 3). Furthermore, small bowel loops were distended with mucosal fold thickening, representing mild enteritis in the jejunum.

On postoperative Day 7, the patient had flatus and her first bowel movement was minimal in quantity. She was started on a clear liquid diet, discharged, and advised to start a soft food diet in two days and follow up at the outpatient clinic.

## Discussion

LAGB is a minimally invasive bariatric procedure first introduced in 1993. It has since been revised and modified multiple times in an attempt to reach a level of refinement that is seen today [9]. It is praised for its ability to deliver desired results while maintaining alimentary canal continuity. Despite its illustrious perception, LAGB, like many other bariatric procedures, predisposes the patient to an increased risk of life-threatening complications [10].

Although the short-term complication rates associated with LAGB are low, long-term complication rates may be as high as 30-70% [9,11]. The most common complications reported in recent literature include band erosion (2-9%), band intolerance (6.7%), port or band infection (1-4%), gastric band slippage (1-22%), esophageal dilation (14%), food trapping (2%), pouch dilation (4-9%), and band or port leak (1-4%) [6].

Our patient presented with small bowel obstruction (SBO) as a consequence of connecting tube intertwinement within the mesentery, accompanied by dense adhesions two years post-LAGB placement. This is a rare complication without any corresponding statistical evidence to support the high rarity of SBO following LAGB [12]. A previously published Australian report from 2016 described the case of a 52-year-old woman presenting with SBO two years after LAGB placement, which was only the fifth documented case in the country [13]. The case described a closed-loop jejunal obstruction as a result of adhesions to the connecting tube. Another study detailed the case of a 42-year-old woman with SBO who also had adhesions complicated further by bowel perforation 19 months after LAGB placement due to the connecting tube looping around the mesentery [14]. In the majority of the cases, the development of SBO post-LAGB placement results from either adhesions or the free movement of the connecting tube itself within the abdominal cavity [15]. Relative to other complications, SBO post-LAGB placement, although rare, is a potentially life-threatening complication. Thus, modifications to the connecting tube placement, location, and length may provide a viable solution to preventing this development.

Due to our patient’s presentation, exploratory laparoscopy was the initial management in order to alleviate the obstruction, which was initially thought to be due to adhesions. The procedure soon evolved into an exploratory laparotomy as the cause of obstruction remained unknown. The procedure was meticulously executed as extensive adhesiolysis was performed, and LAGB continuity was established without compromise. Prompt surgical intervention was key to the prevention of small bowel ischemia and perforation as was previously seen in other cases with a similar presentation. It is possible that the length of the connecting tube may have a crucial role to play in the development of SBO post-LAGB placement, as was the case with our patient. Due to the physical nature of the LAGB, patients presenting with SBO post-LAGB should not be subjected to traditional SBO management and should be tended to with the utmost due diligence in order to prevent possible life-threatening outcomes.

As an increasing number of patients undergo this procedure, it is crucial to establish a comprehensive guideline for possible procedure-related complications. Such a protocol in addition to statistical significance can help guide management. Clinician awareness of such complications in combination with patients’ detailed surgical history can allow for comprehensive and efficient patient care. By highlighting the LAGB placement-induced SBO, our case aims to bring awareness to future avenues of research.

## Conclusions

SBO secondary to a LAGB procedure is a rare complication of this otherwise low-risk bariatric procedure. The development of adhesions after any surgical procedure is inevitable in most cases. The causative mechanisms behind their development have not been explicitly defined. Despite adhesion development being the leading cause of SBO, the cause of SBO in this patient developed as a direct consequence of excessive LAGB connecting tube length, which leads to its intertwinement within small bowel mesentery. The complication of SBO, although common, can quickly follow fatal sequelae if not expeditiously diagnosed. Thus, emergency personnel and surgeons that encounter SBO in a patient with a history of LAGB placement should be made aware that this common complication may be the result of a rare process, as seen in our patient. The length of the LAGB connecting tube should be taken into consideration by surgeons when the device is initially placed in order to minimize the likelihood of developing SBO caused by this rare mechanism. Due to the fact that this is an extremely infrequent cause of SBO with no statistical data on incidence rates, it is of paramount importance for healthcare workers and device manufacturers to be made aware of this cause of SBO following LAGB placement. Our aim is to bring forth a conscious and thorough appreciation of this case, which will consequently pave the way for improved long-term patient outcomes, as well as further the research and development of LAGB devices and the procedures used in their placement.
